# Plant-Based Proteins Have a Flavor Issue. Chemists Can Help with That

**DOI:** 10.1021/acscentsci.6c01502

**Published:** 2026-09-16

**Authors:** Carolyn Wilke

## Abstract

Microbes, enzymes, and synthetic chemistry might help remove bad-tasting molecules in pea and other plant protein

## Graphical abstract

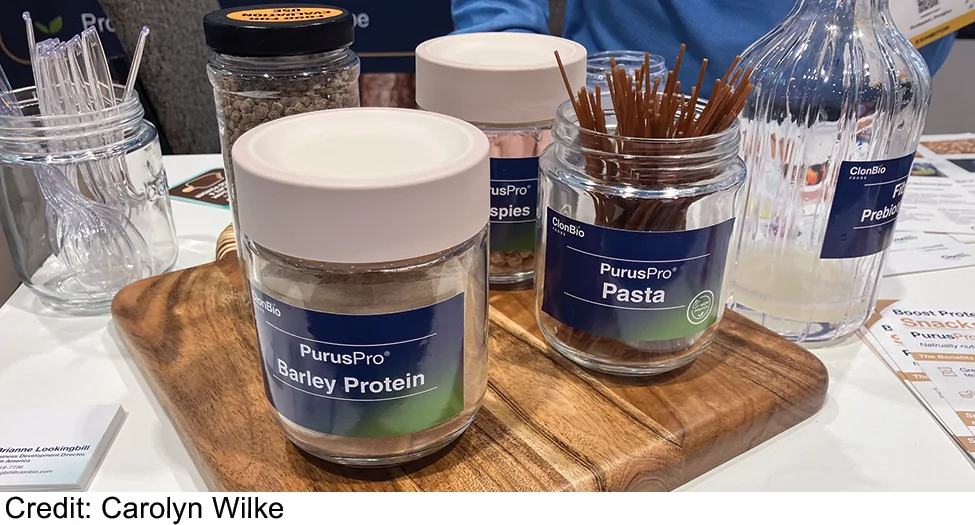

**C** **hicago**—Standing at a booth at IFT FIRST, a food science expo where exhibitors are doling out samples, I take a sip of kuding tea and wince. The concoction is profoundly bitter, thanks to saponins and other compounds. Evelyn Su, founder and CEO of Sino Lion USA, stirs a spoonful of white powder into my cup. I sip again and now detect a subtle sweetness—the bitterness is gone.

**Figure:**
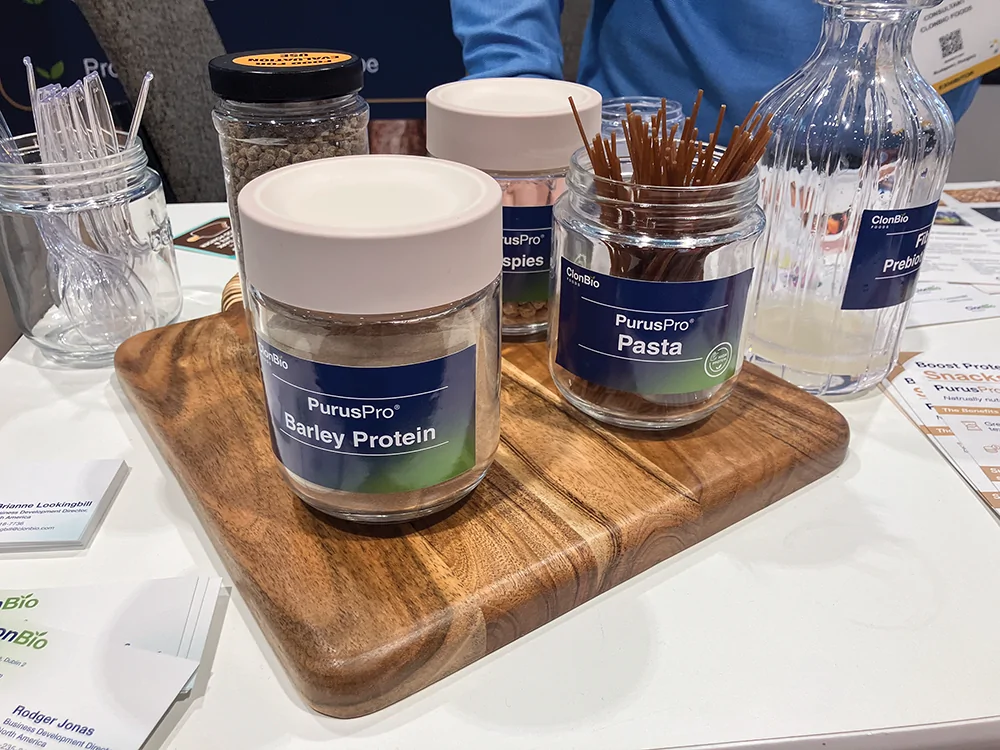
Exhibitors displayed a variety of plant proteins at the 2026 IFT FIRST food science and technology expo, held in Chicago from July 11 to July 15. The ClonBio booth shared samples of food, including pasta, made with the company’s PurusPro barley protein. Credit: Carolyn Wilke/C&EN

“It’s night and day,” Su said. The powder contains ring-shaped molecules called cyclodextrins that can trap foul-tasting compounds. It’s one of the NeverBitter line of ingredients made by an affiliate company and used in products such as sports drinks in China. The team tailors the size of the cyclodextrin rings to capture a given off-putting molecule and prevent it from interacting with a person’s taste buds, Su said. The approach is one of many from food companies and academic researchers that could help make plant proteins more palatable.

Plant proteins often don’t taste good. They can be bitter and come with a whiff of beany or grassy odors. They can even feel yucky. High astringency can cause a sandy sensation on the tongue and make the mouth feel dry. Taste tests of plant proteins including pea, soy, fava bean, lentil, chia, sunflower, and some made from cereal grains have produced unpleasant characteristics that tasters describe as “musty,” “hay-like,” and “soapy.”

Over 2 days at the expo, which is organized by the Institute of Food Technologists, I tried a lemon-and-lime-flavored slushy made with pea protein and collagen, protein powder made from watermelon, pea protein ice cream, and a chocolate-pistachio cake enriched with fava bean protein. I even tasted a coffee creamer touting 2 g of protein from rice, pea, and oats per serving. Some of these things were delicious. Others left an aftertaste.

With diet trends such as “protein-maxxing” driving demand, “animal sources, in particular whey proteins, [have] tripled and quadrupled in price,” said Brian Walker, president of the ingredient company SternMaid America. Whey protein isolates now cost around $30 per kilogram in the US, but fava beans have remained steady at about $14 per kilogram, he said. “Suddenly, people are looking for other protein sources that are more economically viable.” Meanwhile, much of the world lacks quality protein, and there’s a need for more-affordable and more-sustainable protein options.

Costs aside, global food production accounts for about one-third of total greenhouse gas emissions, and raising animals contributes around half of that. Plant-based options could help satisfy the protein demand while placing less strain on the planet—that is, if flavor chemists and food manufacturers can work their magic by eliminating bad flavors and transforming molecules to make plant protein taste good.

## Why Does Plant Protein Taste So Bad?

“It doesn’t matter really the source of that plant protein,” said Clifford Hall, a food scientist at South Dakota State University. “Many of them suffer from the same types of issues.”

Some of these icky tastes stem from compounds that plants make to deter insects. These include saponins—phenolic compounds that are found in all plants and can give a bitter or soapy flavor. These nonvolatile compounds tend to bind to the protein when it is extracted.

Plants also make volatile compounds that influence taste through odor. Lipid oxidation products created by enzymes, such as those that transform fats stored in seeds, can produce aldehydes that smell grassy, or pentanol, which imparts a cardboard-like flavor.

**Figure:**
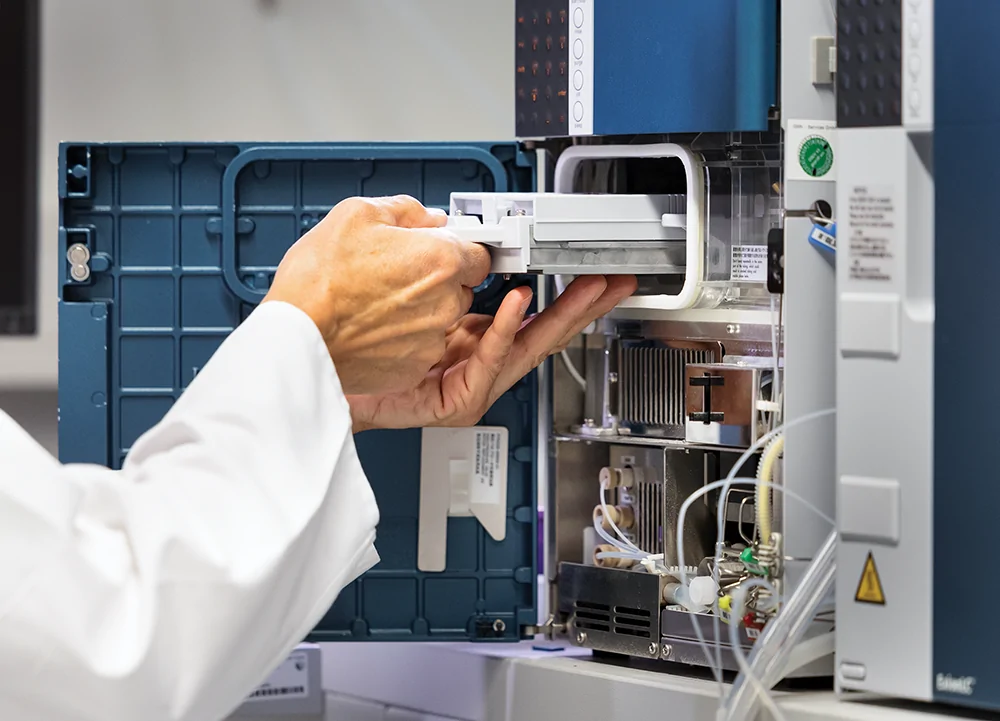
Flavor-focused researchers use a mass spectrometer to study the compounds that contribute bitterness or umami to a protein’s taste. Credit: J. Krpelan/Leibniz, LSB@TUM

One of the most characteristic flavors comes from alkyl methoxypyrazines, which lend green and earthy notes. “Those are the ones that really give that pea-flavor intensity,” Hall said. In 2011, he and colleagues tried to make a protein-packed, gluten-free bread with pea flour. Its flavor was so strong that “it was actually worse than the pea,” he said. They scrapped the idea.

Pea protein, specifically, has three layers of sensory attributes to manage, according to Kushal Chandak, vice president of R&D at Puris, a large pea protein manufacturer. Smell, texture, and, of course, taste: “Almost like pea soupy notes,” he said.

People can try to breed plant varieties that have less of the unpleasing flavor compounds. But it can take breeders over a decade to produce a new variety, and some compounds, such as fatty acids, won’t ever be bred out of plants, said Corinna Dawid, a professor at the Technical University of Munich. At least in the short term, plant proteins need technological solutions.

## Chemically Extracting Bad Flavors

These approaches include using masking compounds, such as cyclodextrins and other often proprietary chemicals. Salt, sugar, and other flavors can also help obscure unpleasant scents and tastes, Dawid said. But companies have other tricks up their sleeves, such as using chemical methods to modify or fish out bad-tasting molecules.

Puris uses pH to control its protein’s properties. First, it disperses the protein in water. It then dials in the pH until the protein reaches a neutral charge and falls out of solution, a process called isoelectric precipitation. As the protein’s charge changes, so does its shape, solubility, thickness, and tendency to foam.

Different processing steps can make proteins suited for other applications. Last year, Puris launched a clear pea protein product. The proteins in it are first chopped into peptides. This cuts the protein content somewhat but makes the ingredient transparent—or nearly so—in water and much thinner than a typical protein powder mixture, which tends to be creamy and is often flavored with chocolate or vanilla.

**Figure:**
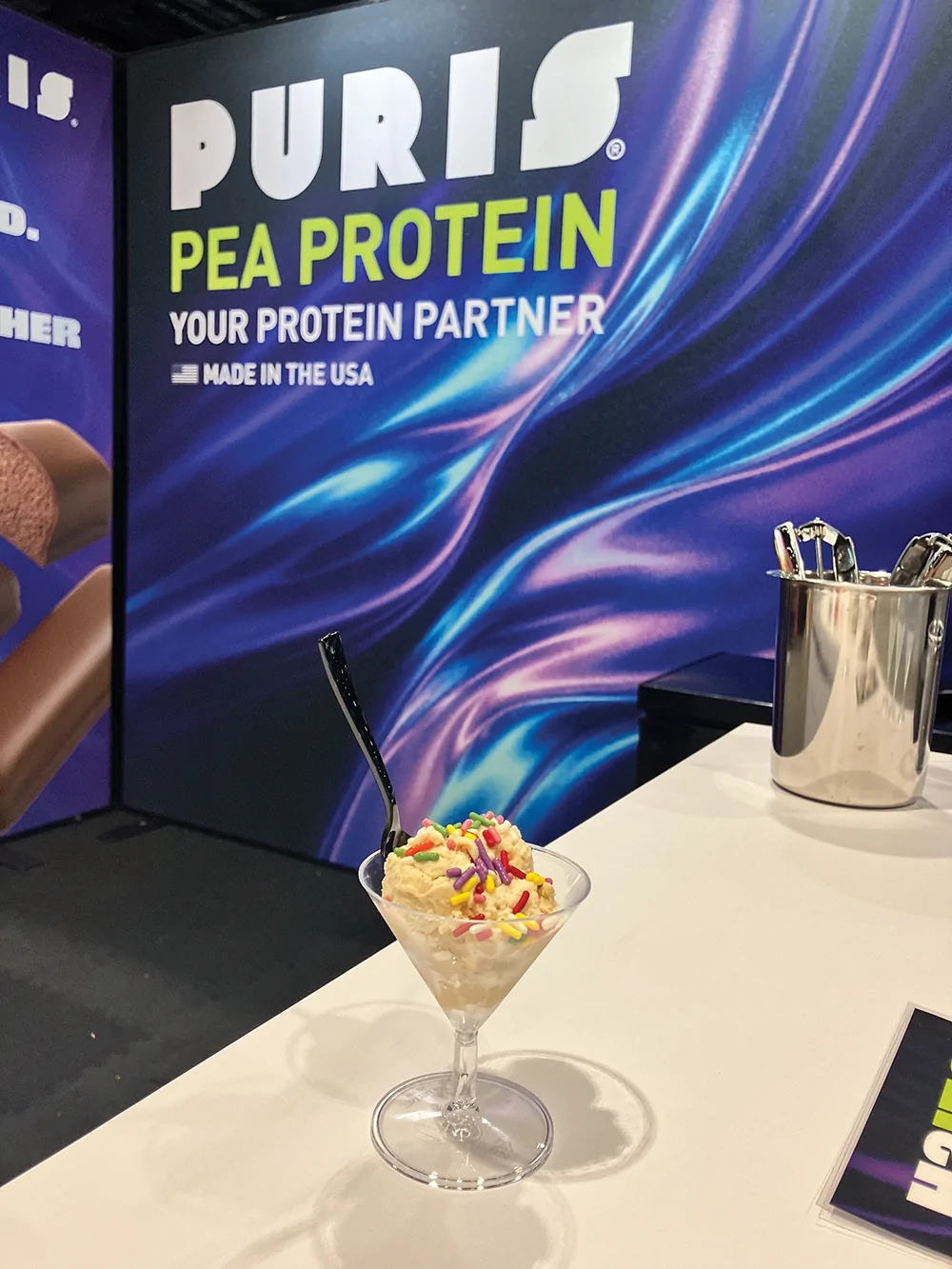
The protein manufacturer Puris provided samples of pea protein ice cream, one of many examples of protein making its way into indulgent foods at the IFT FIRST event in Chicago. Credit: Carolyn Wilke/C&EN

Because clear pea protein is stable in acidic liquids, it can be a better candidate for fruit-flavored sports drinks and protein sodas. “It gives customers a way to utilize protein in a different market segment,” Chandak said.

South Dakota State’s Hall has tested extracting bad-tasting compounds with solvents. The undesirable molecules are often stuck to the plant protein by hydrogen bonding, but that interaction can be disrupted by water or another solvent. “You’re trying to have two compounds compete for one molecule,” Hall said.

His team has gotten the best results and a fairly neutral-tasting pea protein with a supercritical CO_2_ and ethanol extraction. It drastically decreased the total volatiles and reduced the perceived intensity of pea flavor. While supercritical CO_2_ requires expensive equipment, ethanol extraction could easily be added to the production pipeline, Hall said.

Still, solvent extraction is not common for pea protein and is more cost-effective for high-value products like essential oils or spices, Hall said. Food companies are more often using fermentation to improve taste and even give proteins new characteristics.

## Microbes and Enzymes Can Transform Protein

Dawid and her colleagues have searched for suitable strains of microbes for pea protein fermentation. After initially testing dozens of bacteria, the team landed on a *Lactobacillus johnsonii* strain and a *Limosilactobacillus fermentum* strain that contributed amino acids, nucleotides, and peptides that boosted beverages’ umami flavors and saltiness. Follow-up work on *L. johnsonii* identified the key genes in the strain’s protein-cutting ability.

“Our final goal is to identify something which is amazing and new,” rather than mimic the flavor and texture of an animal product, Dawid said. And if the aim is a new type of fermented food, adding flavor-modulating microbes into a fermentation step wouldn’t result in much extra cost, she said.

Free enzymes could be used in addition to microbial fermentation. Vanessa Small, founder and CEO of the start-up Alcheme Bio, said enzymes could target bad flavors in a way that masking can’t. Covering up flavors has been able to get us only so far, she said. “The appetite is much bigger for new solutions, because people have been used to masking for so long.”

Alcheme Bio makes enzymes that transform bad-tasting compounds by targeting functional groups like ketones and aldehydes. For instance, Small said, you could turn an aldehyde into an alcohol for a more neutral taste. She and her team have worked on pea proteins and fermented soy pulp and have reduced astringency, bitterness, and some beany notes by 25–30%, Small said.

## Proteins That Make Your Mouth Pucker

Flavor is just one aspect of eating. Plant proteins don’t just taste bad but often feel bad, too.

The dry mouthfeel caused by plant proteins comes from their properties and interactions with the tongue and saliva, said Anwesha Sarkar, a food scientist at the University of Leeds. Plant proteins are more hydrophobic than many animal proteins we consume. In water, the former tend to cling to each other and form big particles that our mouths perceive as powdery.

In a study of cells that mimic the inside of the mouth, Sarkar’s team found that plant proteins stripped the cells of their mucins—lubricating proteins found in saliva. Animal proteins, such as whey, don’t precipitate salivary proteins in the same way.

Understanding and quantifying these tactile sensations presents a challenge, because it’s not clear which receptors activate the reactions, Sarkar said. So her team studies the interaction of animal proteins surfaces that model the tongue, including a 3D-printed imitation.

The device mimics the real thing in texture, topography, softness, and surface properties. The researchers find that friction and particle sizes can help explain astringency.

Sarkar and colleagues are using their understanding of these mouth sensations to make proteins that are less puckery. They’ve cross-linked proteins and turned them into hydrogels, then chopped down the hydrogels into microgels. “Essentially, you’re creating a hydration sheet around the material,” Sarkar said. The result is a low-friction material that can lend a creaminess to foods like chocolate and yogurt. Her company, MicroLub, is working on such protein replacements for fats and oils.

## Why People Want More Plant Protein

At another booth at IFT FIRST, I sampled a slushy and some desserts containing protein from lupin, a legume used in South America. As I munched a fairly tasty cookie at National Cortina’s booth, a test chef told me that the flour he used was 60% protein (by comparison, wheat flour is about 10–15% protein) and that he thought lupin protein would spread through the market once one of the big food companies picks it up.

The protein-maxxing trends at the expo echo those in society. “This boom in proteins really hit—particularly in the US—with the GLP influence,” SternMaid’s Walker said. With smaller appetites, people taking GLP-1 (glucagon-like peptide 1) drugs often aim for high nutrient density in foods.

Price is another factor for consumers—who are watching whey protein prices skyrocket—and producers alike. Making plant proteins could be economical even with the costs involved in improving flavors. Walker said masking ingredients are expensive per kilogram but are used in relatively small quantities. And Alcheme Bio’s Small said flavor ingredients and processes account for 5–8% of the cost of a plant protein product, while a suite of enzymes provided by her company would be an estimated 1–2%. “It’s a cost savings,” she said.

Still, Walker said, consumers largely perceive plant-based foods as “overpromised and underdelivered.” For instance, some meat alternatives were hits, but other products that weren’t market ready ruined the image of plant-based foods. “To capture the mainstream market, we’ve got to do a lot more,” he said. “We’ve got to offer good-tasting, nutritional products with the right texture and the right flavor and the right cost.”

I’m one of those consumers who could be swayed. Wanting to add more protein into a largely vegetarian diet, I tried an unflavored pea protein isolate around a decade ago. That gag-inducing experience largely turned me off from plant proteins.

“If we can convert a fraction of those people who had that bad experience,” Small said, “it will change the industry.”

As I finish writing this story, I tear open a sample packet of mango pineapple–flavored clear pea protein that I picked up at the expo. I mix it with water in a shaker and taste it. Not bad, I think. I probably could drink this again.

*Carolyn Wilke is a freelance contributor to* Chemical & Engineering News *, the independent news outlet of the American Chemical Society.*

